# The presence of wild type p53 in hematological cancers improves the efficacy of combinational therapy targeting metabolism

**DOI:** 10.18632/oncotarget.4653

**Published:** 2015-07-30

**Authors:** Nerea Allende-Vega, Ewelina Krzywinska, Stefania Orecchioni, Nuria Lopez-Royuela, Francesca Reggiani, Giovanna Talarico, Jean-François Rossi, Rodrigue Rossignol, Yosr Hicheri, Guillaume Cartron, Francesco Bertolini, Martin Villalba

**Affiliations:** ^1^ INSERM U1183, Université de Montpellier 1, UFR Médecine, Montpellier, France; ^2^ Laboratory of Hematology-Oncology, European Institute of Oncology, Milan, Italy; ^3^ Département d'Hématologie Clinique, CHU Montpellier, Université Montpellier 1, Montpellier, France; ^4^ Laboratoire Maladies Rares: Génétique et Métabolisme (MRGM), Université de Bordeaux, Bordeaux, France; ^5^ Cellomet, Amélie Rabat-Léon, Bordeaux, France; ^6^ Institute for Regenerative Medicine and Biotherapy (IRMB), CHU Montpellier, Montpellier, France

**Keywords:** metabolism, oxidative phosphorylation, dichloroacetate, mutant p53, AMPK

## Abstract

Manipulation of metabolic pathways in hematological cancers has therapeutic potential. Here, we determined the molecular mechanism of action of the metabolic modulator dichloroacetate (DCA) in leukemic cells. We found that DCA induces the AMP-activated protein kinase (AMPK)/p53 pathway with increased efficacy in tumors expressing wild type (wt p53). Clinically relevant, low concentrations of doxorubicin synergize *in vitro* and *in vivo* with DCA to further enhance p53 activation and to block tumor progression. Leukemia cell lines and primary leukemic cells containing mutant p53 are resistant to the above-described combination approach. However, DCA synergized with the Hsp90 inhibitor 17-AAG to specifically eliminate these cells. Our studies strongly indicate that depending on the p53 status, different combination therapies would provide better treatment with decreased side effects in hematological cancers.

## INTRODUCTION

Hematological malignancies, such as leukemia, lymphoma and myeloma, affect blood, bone marrow and lymph nodes. Although recent treatments have significantly improved patient survival, many patients will not respond to treatment or will develop resistance. Therefore, novel therapeutic approaches are required [1].

Most cancer cells use elevated amount of glucose for anabolic reactions and generate ATP through aerobic glycolysis rather than via mitochondrial oxidative phosphorylation (OXPHOS). This specific tumor cell metabolism, which is called the Warburg effect [2], provides growth advantages besides its role in anabolism [3] and is also observed in leukemic cells of different origins [4, 5]. Dichloroacetate (DCA), an inhibitor of mitochondrial pyruvate dehydrogenase kinase 1 (PDK1), can shift cellular metabolism from glycolysis to glucose oxidation in a variety of cell lines, including leukemic cells [6]. PDK1 inhibition activates pyruvate dehydrogenase (PDH), a key regulator of glucose oxidation metabolism, causing a reduction in lactate production and an increase in OXPHOS. DCA has been used in medicine for over 30 years to treat metabolic disorders, including lactic acidosis [7, 8]. Furthermore, DCA has shown clinical efficacy for the treatment of glioblastoma [9] and complete remission of a patient with non-Hodgkin's lymphoma was reported after self-administration of DCA [10]. However, the mechanisms responsible for DCA-induced tumor regression have not been clearly elucidated and the effect of DCA in different tumors is still controversial [10–16].

The tumor suppressor gene *p53* is emerging as an important regulator of metabolic homeostasis. It promotes OXPHOS and inhibits glycolysis and thus might hinder the Warburg effect in many cancers [17]. p53 stimulates metabolism by inducing the expression of different metabolic genes, such as cytochrome c oxidase 2 (*SCO2*), glutaminase 2 (*GLS2*), p53 up-regulated modulator of apoptosis (*PUMA*), glucose transporter 1 and 4 (*GLUT1* and *GLUT4*) and TP53-induced glycolysis and apoptosis regulator (*TIGAR*) [18]. p53 also increases *AMPKβ1* and *AMPKβ2* gene expression, reinforcing the AMP-activated protein kinase (AMPK) response [19]. AMPK, the main metabolic cell sensor, is activated in conditions of energetic stress that deplete the cell ATP supplies, such as nutrient deprivation, or in response to oxidative stress caused by hypoxia [20]. AMPK also phosphorylates and stimulates p53 transcriptional activity to initiate a metabolic cell cycle checkpoint [21]. Their mutual regulation enhances their tumor suppressive functions.

More than half of all human tumors harbor mutations in the *p53* gene that abrogate its DNA binding and transactivation activity [22]. Substantial evidence indicates that mutant p53 gain-of-function activity is dependent on its *de novo* ability to activate gene expression [23, 24]. Recently, it has been shown that mutant p53 can bind to the AMPKα subunit and inhibit AMPK signaling in head and neck cancer cells [25]. In hematological malignancies, p53 mutations are less frequent (10–15%) than in solid tumors, but are strongly associated with poor survival, refractory disease and chemo-resistance [26–29]. Moreover, p53 mutation rate increases during disease progression and also in response to chemotherapy. There is growing interest in the role of mutant p53 in tumor invasion and metabolism because it can promote tumor cell proliferation and might suppress other activities of wild type (wt) p53, such as cell respiration and anti-oxidant response.

Hence, targeting cell metabolism, for instance with DCA, could be a new promising strategy for treating hematological cancers [1]. DCA effects in B-chronic lymphocytic leukemia (B-CLL) depend on p53 status [30, 31], probably because DCA activates p53 at post-transcriptional levels [31]. DCA also exhibits toxicity against B-CLL cells lacking wt p53 [30]. However, how DCA activates wt p53 is unknown. Here, we show that targeting tumor metabolism using DCA could be a new effective approach for the treatment of several hematological cancers and that its efficacy depends on the tumor p53 status. DCA, through AMPK phosphorylation, increases p53 transcriptional activity and leads to p53-dependent G_1_ cell cycle arrest. Moreover, p53 activates AMPK through a positive feedback loop. We also show that combination of DCA with genotoxic drugs, such as doxorubicin and vincristine, can greatly improve DCA effectiveness by further promoting activation of wt p53. This could allow reducing the concentration of these drugs to minimize their side effects. We also found that associating 17-Allylamino-17-demethoxygeldanamycin (17-AAG), a heat-shock protein (HSP) 90 inhibitor, with DCA potentiates the apoptotic effect in leukemic cell lines and primary tumor cells with mutant p53. Therefore, this study provides two protocols for DCA-based combinational therapy in hematological cancers based on their p53 status.

## RESULTS

### DCA promotes p53 transcriptional activity and causes cell cycle arrest in a p53-dependent manner

**Figure 1:**
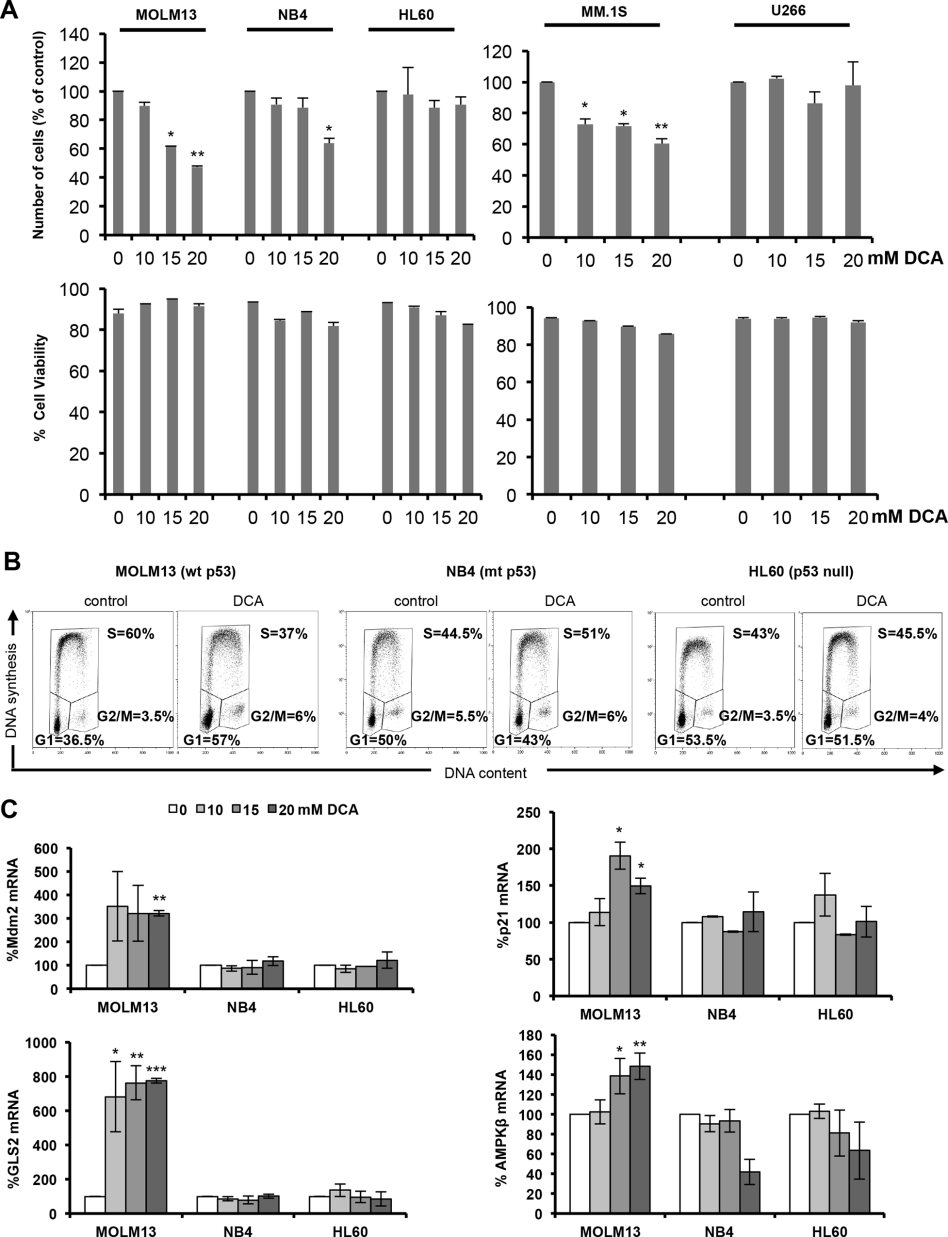
DCA activates p53 and causes p53-dependent cell cycle arrest **A.** AML (MOLM13, NB4 and HL60) and MM (MM1.S and U266) cells were incubated with the indicated concentration of DCA for 48 h and then cell viability and alive cell numbers were determined using the Muse^®^ Cell Analyzer. Data are means ± SEM of two independent experiments **B.** DCA causes G1 cell cycle arrest in leukemic cells harboring wt p53. MOLM13 (wt p53), NB4 (mutant p53) and HL60 (p53−/−) cells were cultured in the presence of 20 mM DCA for 48 h. Cells were then pulse-labeled with EdU and harvested for cell cycle distribution analysis (wt, wild type; mt, mutant). **C.** mRNA levels of the p53 target genes *MDM2*, *p21*, *GLS2* and *AMPKβ*. Values are the mean ± SEM of results from two independent experiments performed in triplicate in cells incubated with the indicated DCA concentrations for 24 h.

We previously showed that DCA, a small molecule that inhibits PDK1 (a key regulator of the Warburg effect), blocks aerobic glycolysis in leukemia cells [6]. Here, we examined DCA effect on growth and viability of three acute myeloid leukemia (AML) cell lines (MOLM13, NB4 and HL60) and in two multiple myeloma (MM) cell lines (MM1.S and U266) with different p53 status (Supplementary Table S1). After 48 hours of incubation with increasing concentrations of DCA, the number of cells was significantly reduced, in a dose-dependent manner, in MOLM13 and MM1.S cells (wt p53), but not in U266 cells (mutant p53) or in HL60 cells, in which p53 was genetically ablated (p53−/−). In NB4 cells (mutant p53), the cell number was reduced only upon incubation with the highest DCA concentration. Cell viability was not inhibited in any of the cell lines under study (Figure 1A). We next investigated DCA effect on the cell cycle by incubating the three AML cell lines with 20 mM DCA for 48 hours. Cell cycle distribution analysis showed that following DCA treatment, the proportion of MOLM13 cells (wt p53) in G1 was increased and the percentage of cells in S phase was reduced compared to untreated cells (Figure 1B). Conversely, in NB4 and HL60 cells progression to S phase was not suppressed by DCA. These results indicate that DCA induces G1 cell cycle arrest and blocks cell proliferation in a p53-dependent manner. Accordingly, DCA induced p53 transcriptional activity only in MOLM13 cells (Figure 1C), leading to up-regulation of p53 target genes involved in cell cycle arrest (*p21*), regulation of p53 activity (*MDM2*) and regulation of metabolism (*GLS2* and *AMPKβ1*). Lower DCA concentration (1–5 mM) could also increase Mdm2 and GLS2 mRNA levels, however we found that high concentration of DCA (10–20 mM) are required to induce a robust p53 response in AML cell lines (Supplementary Figure S1). In summary, in AML cells harboring wt p53, DCA activates p53 transcriptional activity, leading to *p21* up-regulation and cell proliferation inhibition. Moreover, the increased expression of metabolic regulators, such as *GLS2* and *AMPKβ*, indicates that DCA also activates the p53-regulated metabolic response.

### p53 is activated by AMPK in response to DCA treatment

**Figure 2:**
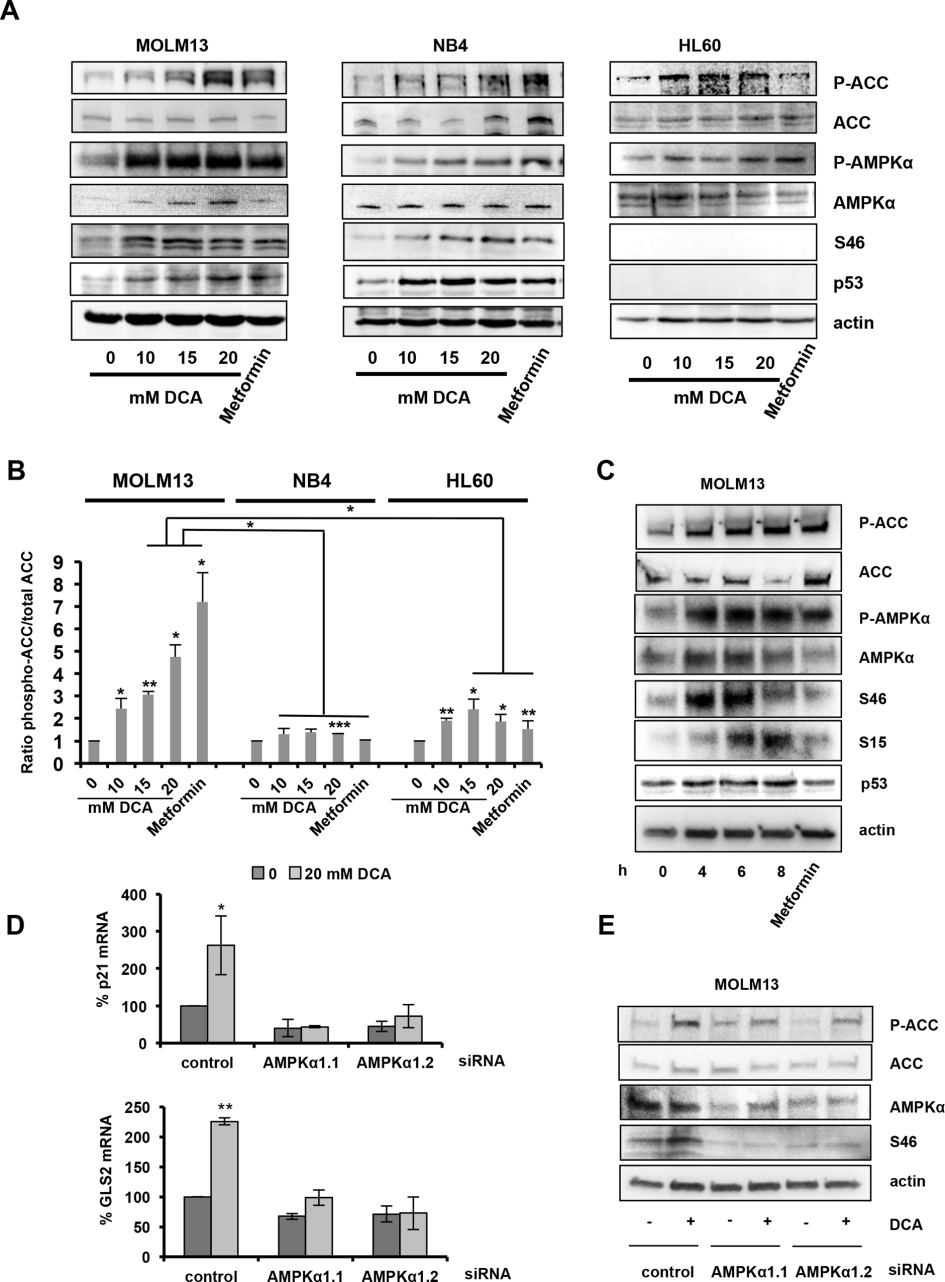
DCA-mediated activation of the AMPK pathway is required for p53 induction **A.** AML cells were incubated with the indicated concentrations of DCA or 5 mM metformin for 24 hr. DCA increases the phosphorylation levels of ACC (at Ser79), AMPKα (at Thr172) and p53 (at Ser46). **B.** Graph representing the ratio between phosphorylated ACC and total ACC protein levels. Bands were quantified using the Image lab software from two independent experiments. **C.** Time-course analysis by western blotting of the effect of 20 mM DCA in MOLM13 cells, including p53 phosphorylation on Ser15 and Ser46. **D.** AMPKα1 silencing with two different siRNAs attenuates DCA-induced p53 transcriptional activity in MOLM13 cells. Quantitative RT-PCR analysis of *p21* and *GLS2* mRNA levels (values are the mean ± SEM of two independent experiments performed in triplicate). **E.** AMPKα1 silencing decreases DCA-induced phosphorylation of ACC (Ser79) and p53 (Ser46). See also Supplementary Figure S2.

These data are consistent with previous studies in solid tumors showing that DCA activates the p53 pathway [9, 31, 32]; however, the underlying mechanism is unclear. As activation of AMPK, the main cellular metabolic sensor, leads to p53 up-regulation and G1 cell cycle arrest [21], we asked whether AMPK could be involved in DCA effects in wt p53 AML cells. Similarly to the AMPK activator metformin [33, 34], DCA increased phosphorylation of AMPKα at the activating residue T172 and also phosphorylation, and thus inactivation, of acetyl-coA carboxylase (ACC), the main AMPK substrate (Figure 2A). ACC phosphorylation was significantly higher in MOLM13 cells (wt p53), than in NB4 (mutant p53) and HL60 (p53−/−) cells (Figure 2B). In the MM cell lines, we also observed a significant increase of ACC phosphorylation in MM1.S (wt p53) compared to U266 (mutant p53) at 10 and 20 mM DCA concentrations (Supplementary Figure S2A and S2B). Moreover no increase in p53 and p21 protein levels was detected in U266 in contrast to MM1.S (Supplementary Figure S2A and S2B).

Activated AMPKα phosphorylates p53, but the sites of this phosphorylation are unknown [21, 35]. DCA reproducibly increased p53 expression and promoted p53 phosphorylation at S46 (Figure 2a and Supplementary Figure S2) and S15 (Figure 2C). Moreover, time course analysis of the effects of 20 mM DCA indicated that p53 phosphorylation at S46 preceded phosphorylation at S15 in MOLM13 cells (Figure 2C).

**Figure 3:**
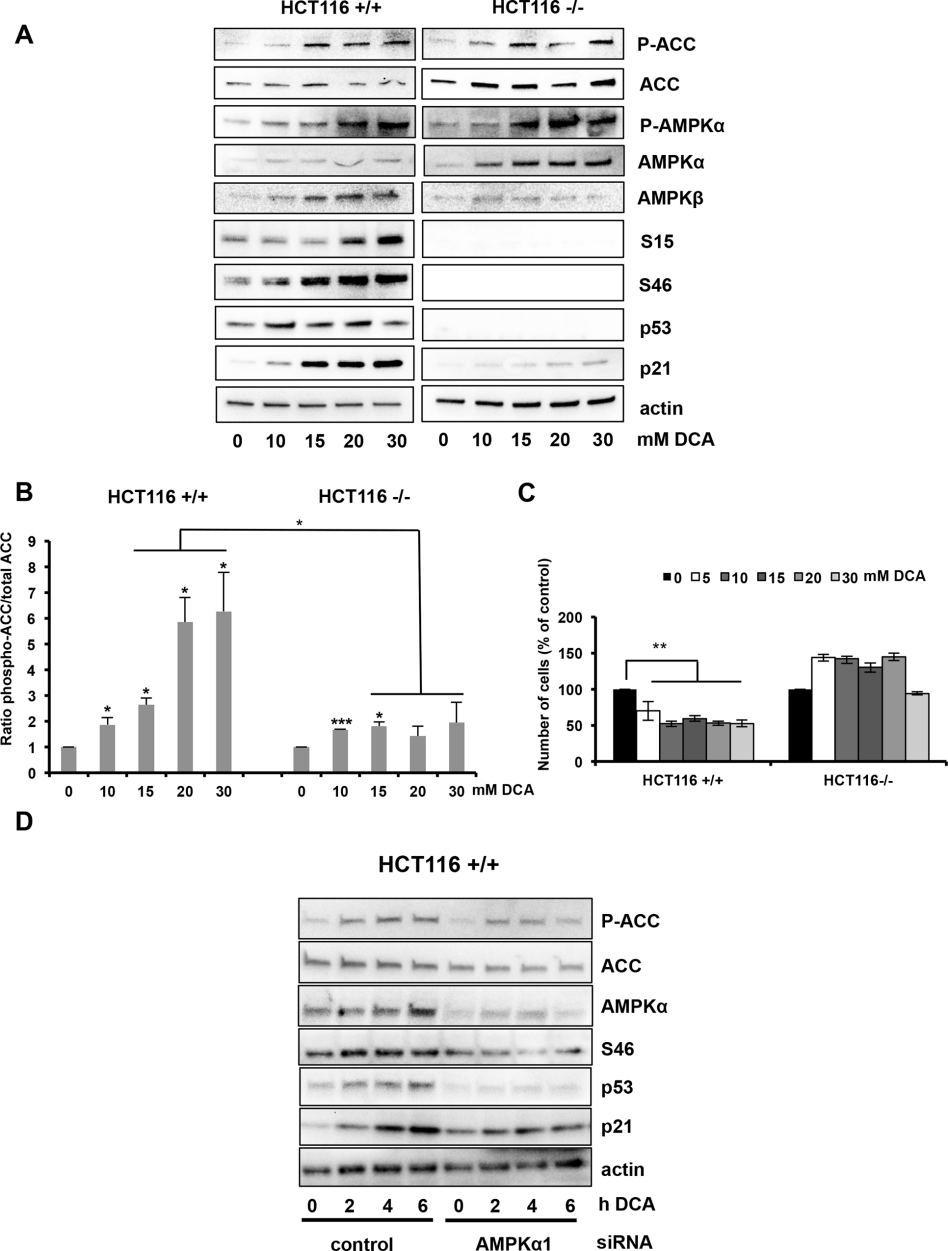
Activation of the AMPK/p53 pathway by DCA in HCT116 cells **A.** Western blot analysis shows the protein levels of phosphorylated ACC, phosphorylated AMPKα, phosphorylated p53 (Ser15 and Ser46), total AMPKα, AMPKβ, p53 and p21 in p53^+/+^ and p53^−/−^ HCT116 isogenic cells after incubation with DCA at the indicated concentrations for 24 h. **B.** Quantification of ACC phosphorylation from the western blot experiments from two independent experiments. **C.** HCT116 cells were incubated with increasing concentrations of DCA for 48 h and then the number of cells was evaluated by using the Muse^®^ Cell Analyzer. Data are means ± SEM of two independent experiments **D.** *AMPKα1* silencing inhibits p53 accumulation and phosphorylation in response to DCA. p53^+/+^ HCT116 cells were transfected with control siRNA and the siRNA against *AMPKα1*. 72 h later they were incubated or not with 20 mM DCA for 2, 4 and 6 hours and then protein expression was analyzed by western blotting.

The different results obtained using wt and mutant p53 AML and MM cell lines could be due to their different genetic background. However, similar results were obtained also using two isogenic colon cancer cell lines (p53^+/+^ and p53^−/−^ HCT116 cells) (Figure 3A). DCA induced higher ACC phosphorylation in p53^+/+^ than in p53^−/−^ HCT116 cells (4.75- and 1.49-fold, respectively; Figure 3A and 3B). Low DCA concentrations induced p53 phosphorylation on S46, whereas relatively higher doses were required to detected p53 phosphorylation at S15 in p53^+/+^ HCT116 cells (Figure 3A). Moreover, DCA inhibited proliferation of p53^+/+^ but not of p53^−/−^ HCT116 cells (Figure 3C). Finally, DCA-induced AMPKβ up-regulation was p53-dependent (Figure 3A), as observed for the mRNA expression (Figure 1).

As DCA promotes reactive oxygen species (ROS) production [16] and an increase in intracellular ROS levels might activate AMPK [20], we measured ROS production in AML cells before and after incubation with DCA. Both NB4 (p53 mutant) and HL60 (p53−/−) cells had higher basal ROS levels than MOLM13 (wt p53) cells (Supplementary Figure S3A, upper panels), possibly due to the lack of p53 anti-oxidant function [36]. DCA treatment stimulated ROS production in a time-dependent manner in all AML cell lines, as determined by quantification of CM-H_2_DCFDA fluorescence, but the increase was higher in MOLM13 cells (Supplementary Figure S3A). AMPK-mediated activation of p53 should contribute to the cell anti-oxidant defenses by inducing the expression of several genes involved in the anti-oxidant response, such as *GLS2* [37–39]. Indeed, DCA incubation led to *GLS2* mRNA up-regulation only in MOLM13 cells (wt p53), but not in p53 mutant cell lines (Figure 1C). These data suggest that DCA-induced ROS production could be responsible for the activation of the AMPK/p53 pathway. To verify this hypothesis, we analyzed the effect of blocking ROS production by *N*-acetyl cysteine (NAC) on DCA-induced AMPK/p53 pathway (Supplementary Figure S3B). NAC treatment completely reversed DCA-induced ROS production and significantly attenuated DCA-induced p53 transcription activity (Supplementary Figure S3C). Moreover, western blot analysis indicated that DCA-induced phosphorylation of p53 (S46), AMPK and ACC was reduced or abrogated by NAC (Supplementary Figure S3D).

To further investigate whether DCA induces p53 activation via AMPK, we silenced AMPKα1 in MOLM13 and p53^+/+^ HCT116 cells using two specific siRNAs. AMPKα1 down-regulation reduced DCA-induced ACC phosphorylation and p53 phosphorylation at S46 (Figure 2E and 3D). Moreover, it inhibited p53 transcriptional activity in both cell lines (Figure 2D and 3D). These data indicate that DCA induces p53 phosphorylation at S46 and p53 transcriptional activity via AMPK activation. Therefore, in order to confirm the essential role of p53 on DCA-mediated inhibition of proliferation, we utilized siRNA to attenuate p53 expression in p53^+/+^ HCT116 cells (Supplementary Figure S4A and S4B). The knockdown of *p53* expression was demonstrated by quantitative RT-PCR (Supplementary Figure S4B). Knockdown of p53 abrogates the effect of DCA on proliferation (Supplementary Figure S4A) and transcription (Supplementary Figure S4B). All these results demonstrate that wt p53 is critical for DCA-induced inhibition of cell proliferation.

### OXPHOS activates AMPK

**Figure 4:**
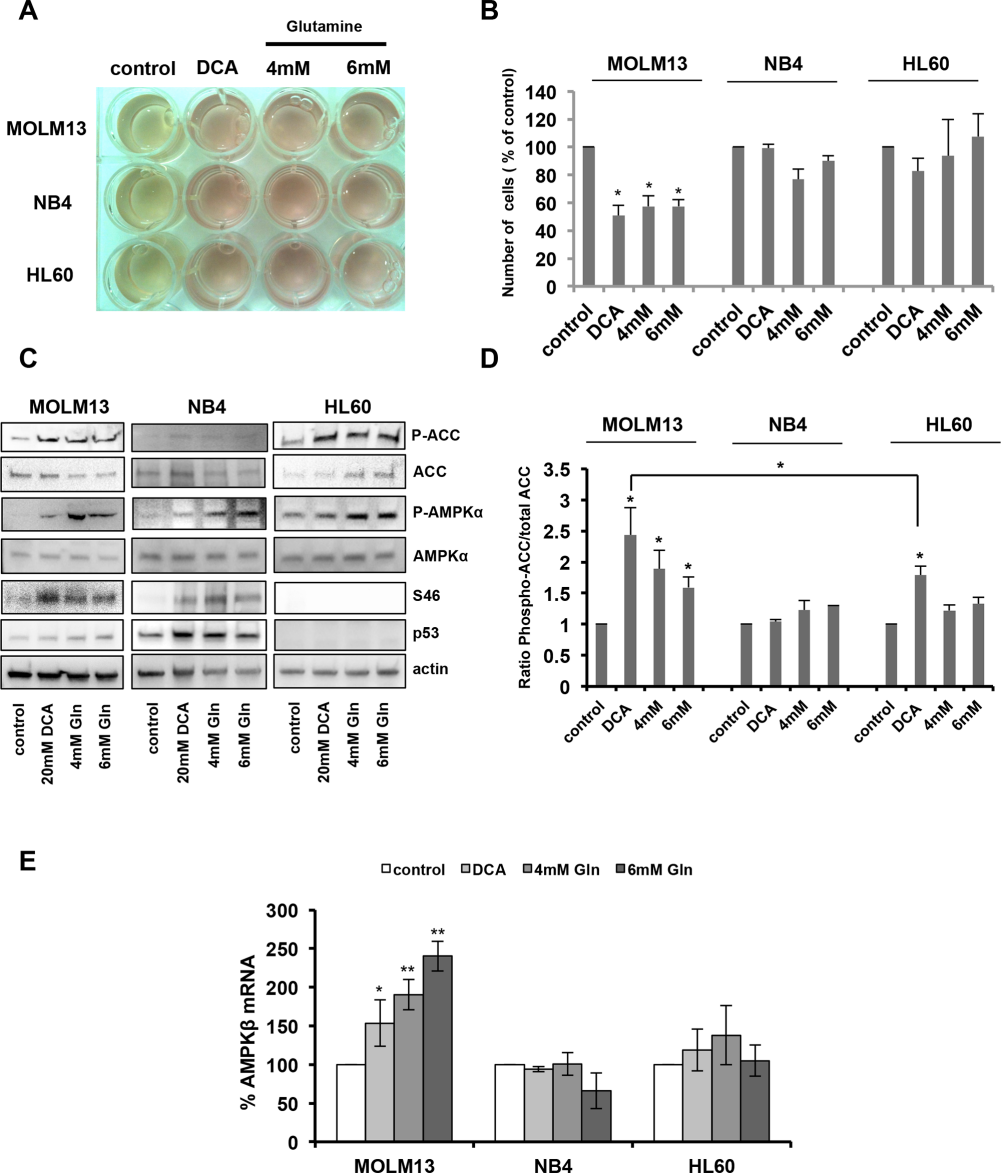
Oxidative phosphorylation activates AMPK and the p53 pathway **A.** AML cell lines were cultured in the presence of 20 mM DCA or in OXPHOS medium (4 mM Gln or 6 mM Gln) for 24 h. The yellow color of the medium indicates lactic acid production. **B.** Growth inhibition of MOLM13 cells incubated with 20 mM DCA or grown in OXPHOS medium (data are means ± SEM of two independent experiments). **C.** Western blotting showing the phosphorylation of ACC, AMPKα and p53 (Ser46) in AML cells grown in OXPHOS medium or in the presence of 20 mM DCA for 24 h. **D.** Quantification of the results from two independent experiments. **E.** RT-qPCR analysis of AMPKβ expression (values are the mean ± SEM of two independent experiments performed in triplicate) in cells treated as in (C).

DCA is a metabolic drug that inhibits aerobic glycolysis (the Warburg effect) and favors the oxidation of pyruvate in mitochondria, fueling OXPHOS. When glucose is no longer available, cells use alternative energy substrates, such as glutamine (Gln). Gln oxidation, or glutaminolysis, generates ATP through OXPHOS and this pathway is functional in leukemic cells [40, 41]. We thus asked whether culturing cells in OXPHOS medium (glucose-free medium supplemented with galactose and Gln [6, 40, 41]) would have similar effects as DCA on cell proliferation and AMPK activation. DCA and OXPHOS medium both blocked lactate production, the end-product of aerobic glycolysis, as indicated by the decrease in acidification of the culture medium (Figure 4A). The reduction in lactate production by DCA is the result of PDK inactivation and Pyruvate Dehydrogenase (PDH) activation and shows that DCA-mediated PDK inhibition is effective in the three AML cell lines. DCA had no effect on oxygen consumption in NB4 and HL60 cells, while it caused a decrease in MOLM13 cells (Supplementary Figure S4C). This indicated that the primary effect of DCA treatment is not a stimulation of oxygen consumption but more a reduction of lactate production. An explanation to the selective effect in MOLM13 cells could be that they stop proliferate and need much less ATP, thereby decreasing oxygen consumption. Indeed, both DCA and OXPHOS medium decreased proliferation in wt p53 MOLM13 cells, but not in p53 mutant NB4 or p53−/− HL60 cells (Figure 4D), indicating that this effect is p53-dependent. Conversely, OXPHOS medium induced AMPK phosphorylation and activation in MOLM13 cells (Figure 4C and 4D). In addition, OXPHOS medium also induced p53 phosphorylation at S46 (Figure 4C) and p53-dependent increase in *AMPKβ1* transcription (Figure 4E). These data suggest that the metabolic change from glycolysis to OXPHOS following incubation with DCA is responsible for the activation of the AMPK/p53 pathway.

### DCA decreases tumor progression in primary leukemic cells

We then validated our results using peripheral blood and bone marrow samples from patients with different hematological malignancies (Supplementary Table S1). First, we used primary tumor cells from a patient with B-cell lymphoma (BCL) (wt p53) that actively proliferate *in vitro* (Patient 2). Alike MOLM13 cells, incubation with DCA for 48 h induced cell cycle arrest in G1 in a dose-dependent manner (Supplementary Figure S5A). In addition, DCA did not inhibit tumor cell viability (Supplementary Figure S5B). Analysis of tumor cells from 31 patients with different hematological malignancies harboring wt p53 (Supplementary Table S1) confirmed that incubation with DCA caused minor effects on cell viability, similar to what observed in CD3^+^ T cells from healthy donors (Supplementary Figure S5C).

**Figure 5:**
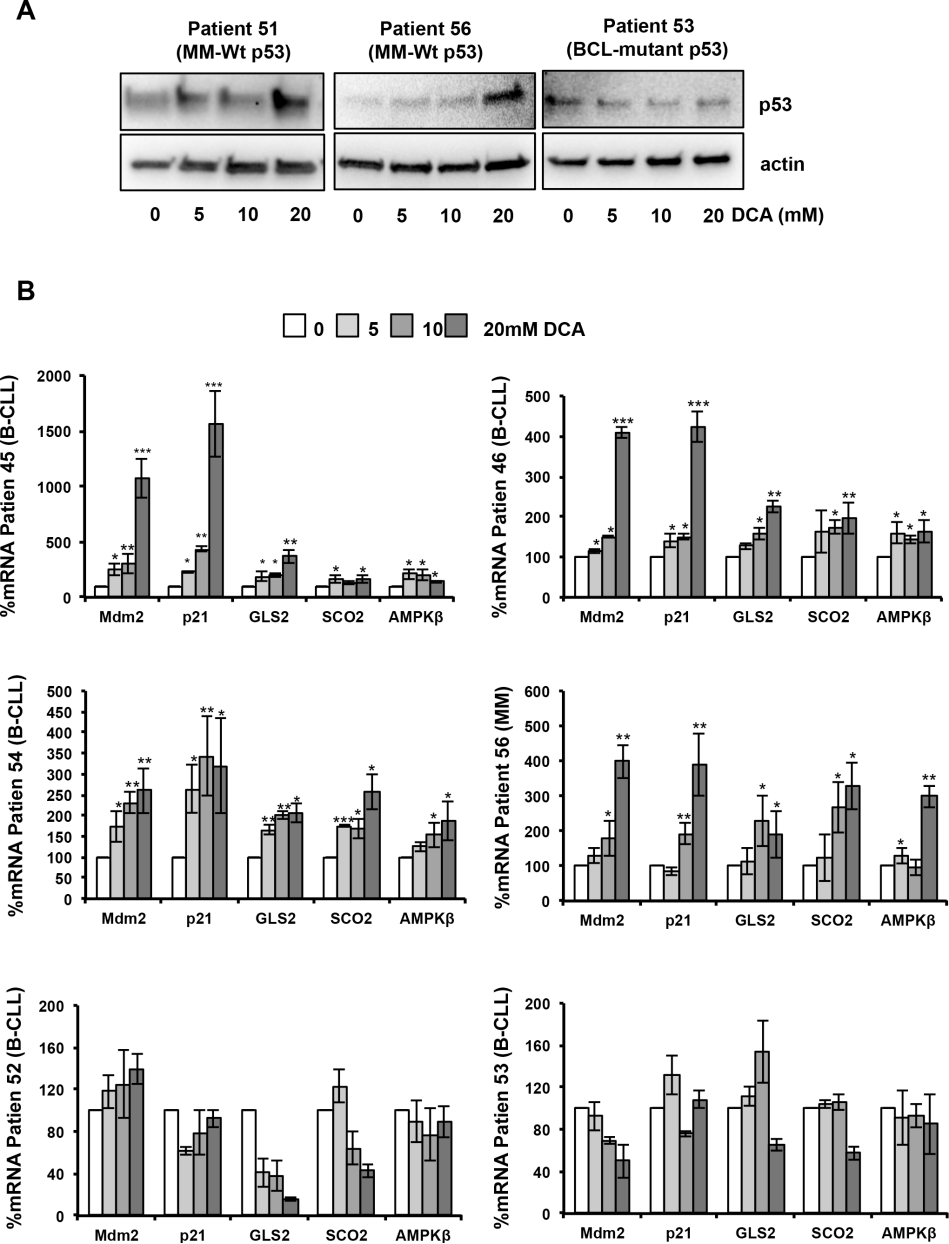
DCA activates the p53 pathway in primary leukemic cells **A.** Accumulation of p53 protein following 24-hour incubation with DCA in tumor cells from patients with MM (Patient 51, Patient 56) harboring wt p53. As control, tumor cells from a patient with BCL carrying mutant p53 are shown. **B.** DCA increases AMPKβ, Mdm2, p21, GLS2 and SCO2 mRNA expression in BCL and MM cells with wt p53 (Patients 45, 46, 54 and 56) after 24 h of treatment, but not in B-CLL cells harboring mutant p53 (Patient 52, Patient 53); *p < 0.05; **p < 0.01; ***p < 0.001. Values are the mean ± SEM from two set of experiments performed in triplicate.

We then examined p53 protein levels and transcriptional activity following DCA incubation in samples from MM, BCL and B-CLL patients (Figure 5). We chose patients with high percentage of tumor blasts, which allowed biochemical studies of rather homogenous cell populations. Expression of p53 protein was increased only in samples from patients harboring wt p53, but not mutant p53 (Figure 5A). Similarly, DCA induced *AMPKβ1* mRNA expression (Figure 5B) and up-regulation of the p53 target genes *MDM2*, *p21*, *GLS2* and *SCO2* (Figure 5B) only in patients' tumor samples expressing wt p53. Therefore, in these patient samples, in addition to the induction of genes related to cell cycle arrest (*p21)*, DCA also stimulated tumor cell metabolism by promoting the expression of *AMPKβ1*, *GLS2* and *SCO2*.

### DCA synergizes with the chemotherapeutic drugs doxorubicin and vincristine

Many chemotherapeutic drugs currently used in the clinic activate the p53 pathway, but also cause DNA damage and many side effects. Combination therapy is a strategy based on the use of multiple chemotherapeutic drugs to efficiently activate p53 with reduced genotoxic effects.

**Figure 6:**
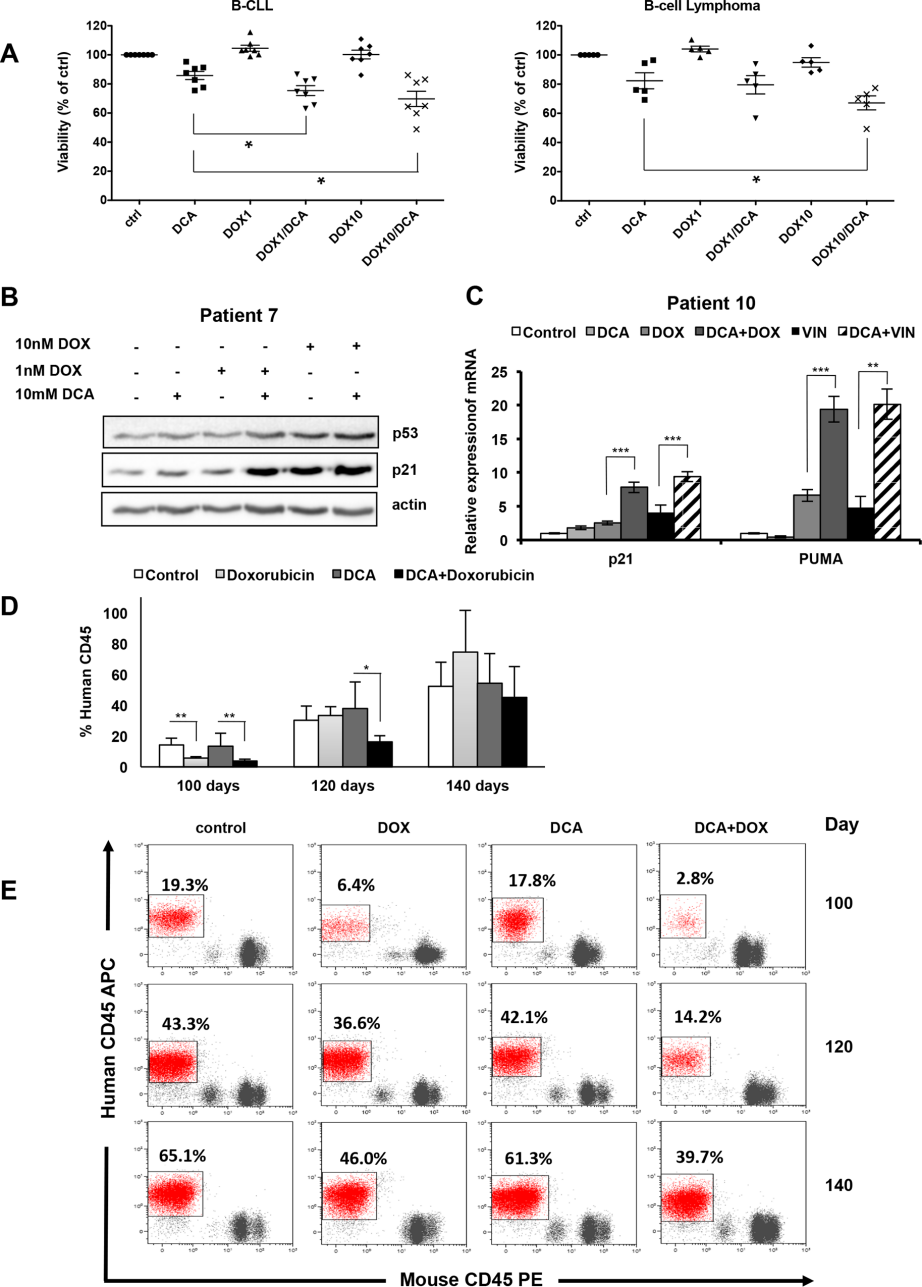
Synergistic anti-proliferating effect of DCA in combination with doxorubicin in primary leukemic cells **A.** Tumor cells from seven patients with B-CLL and five patients with B-cell lymphoma (all wt p53) were incubated with 10 mM DCA alone or in combination with doxorubicin (1 or 10nM; DOX) for 72 h. Tumor cells were identified (CD19^+^/CD20^+^) by flow cytometry and tumor cell death was quantified by 7-AAD staining using the Muse^®^ Cell analyzer (values are the mean ± SEM from one experiment performed in triplicate *p < 0.05). **B.** Tumor cells (wt p53) from a patient with B-CLL (Patient 7) were treated with 10 mM DCA alone or in combination with doxorubicin (DOX) for 24 h. p53 and p21 expression was assessed by western blotting. **C.** mRNA levels of *p21* and *PUMA* in primary tumor cells from a patient with MDS (wt p53; Patient 10) following incubation or not with 10 mM DCA for 24 h and then addition or not of 10 nM doxorubicin (DOX) or 10 nM vincristine (VIN) for another 24 h before analysis by RT-qPCR; **p < 0.01; ***p < 0.001. See also Supplementary Figure S6 (values are the mean ± SEM from one experiment performed in triplicate). **D.** NSG mice were engrafted with primary human AML cells (wt p53). At day 80 post-graft, they were treated with doxorubicin (*n* = 4), DCA (*n* = 4) or both (*n* = 4) and the percentage of human cells in peripheral blood samples was measured every 20 days; *p < 0.05; **p < 0.01 (values are expressed as median ± SEM). **E.** Representative plots of the percentage of human tumor cells in peripheral blood samples of mice at day 100, 120 and 140 post-graft.

We thus tested in leukemic cell lines and primary leukemic cells the anti-proliferative and pro-apoptotic effects of DCA alone or in combination with the chemotherapeutic agents doxorubicin and vincristine. In the AML and MM cell lines harboring wt p53 (MOLM13 and MM1.S), co-treatment with DCA and low doses of doxorubicin or vincristine further reduced the number of leukemic cells in comparison to each drug alone (Supplementary Figure S6A). Conversely, in p53 mutant and p53−/− cell lines both single-drug and combination treatments had little effect on the cell number (Supplementary Figure S6A). Overnight pre-treatment with DCA followed by doxorubicin induced apoptosis in MOLM13 cells (wt p53), but not in NB4 cells (mutant p53) (Supplementary Figure S6B). Similarly, the combined treatment was more effective in reducing tumor cell viability in primary leukemic samples obtained from patients with B-cell chronic lymphocytic leukemia (B-CLL) and B cell lymphoma harboring wt p53 than each single drug (Figure 6A). Co-treatment led to higher accumulation of p53 and of its transcriptional target p21, which controls cell cycle progression (Figure 6B and Supplementary Figure S7A), and also to significantly stronger induction of the pro-apoptotic p53 target gene *PUMA* (Figure 6C and Supplementary Figure S7B). These results show that combination of DCA with non-toxic doses of genotoxic agents could be an approach to induce apoptosis in wt p53 tumor cells.

To test this *in vivo*, we engrafted wt p53 human AML primary cells in non-obese diabetic/severe combined immunodeficient (NOD/SCID)-interleukin-2 receptor γ null (NSG) mice following our previous protocol [42, 43]. Mice with established tumors (day 80 post-graft) were treated with low doses of doxorubicin, DCA or both (Figure 6d and 6e). These treatments were not toxic in non-grafted mice (data not shown). Doxorubicin treatment reduced the proliferation of human AML cells compared to untreated mice (control) only during the first 20 days after the beginning of treatment. Indeed, this effect was not detected at day 120 post-graft (i.e., 40 day after the beginning of treatment). DCA alone did not show any effect. Co-treatment with DCA and doxorubicin significantly delayed tumor progression and the number of circulating human AML cells was still significantly lower compared to control at day 120 post-graft (Figure 6D and 6E). However, sixty days after the beginning of treatment (day 140 post-graft), when mice were sacrificed, we did not observe any difference between groups. Our results show that low doses of DCA and doxorubicin cooperate in blocking proliferation of human AML cells *in vivo*.

### Synergistic effect of DCA with 17-AAG

**Figure 7:**
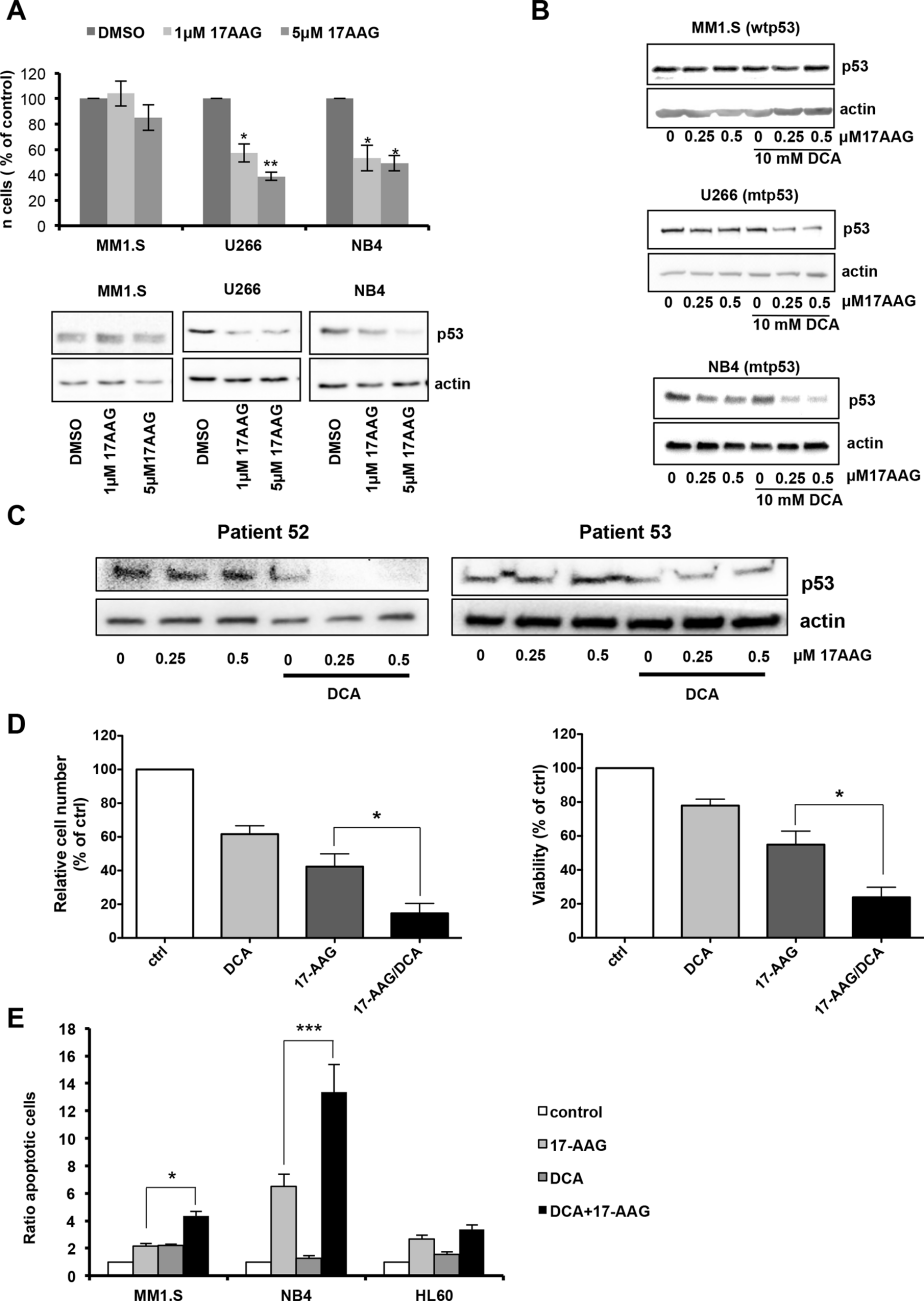
DCA and 17-AAG synergize to induce apoptosis in mutant p53 tumor cells **A.** Effect of 17-AAG in human leukemia cells with different p53 status. MM1.S (wt p53), U266 and NB4 (mutant p53) cells were incubated with 1 μM or 5 μM 17AAG for 24 h. Alive cell number was determined by Trypan blue exclusion. p53 protein levels were analyzed by western blot analysis. Data are means ± SEM of two independent experiments **B.** The combination of DCA and 17-AAG further decreases p53 protein expression in mutant p53 leukemic cell lines. Cells were treated with the different drugs for 24 h before protein expression analysis by western blotting. **C.** Decrease of p53 protein levels in primary tumor cells harboring mutant p53 after co-treatment with DCA and 17-AAG for 24 h. **D.** Effect of DCA alone or in combination with 17-AGG on viability of primary tumor cells. Tumor cells carrying mutant p53 from four different patients with B-CLL were treated with 10 mM DCA and/or 0.5 μM 17-AAG for 72 h. Cells were then stained (CD19^+^/CD20^+^ and 7-AAD) and analyzed by flow cytometry. Living tumor cells were counted using the Muse^®^ Cell analyzer. The mutant p53 status was confirmed by sequencing. Data are means ± SEM of one experiment from four patients performed in triplicate **E.** DCA synergizes with 17-AAG to induce apoptosis mainly in leukemic cell lines that contain mutant p53. Cells were incubated with 10 mM DCA and/or 0.5 μM 17-AAG for 24 h and apoptosis was evaluated by Annexin V assay. Data are means ± SEM of two independent experiments.

Mutations in p53 are correlated with increased drug resistance of tumor cells, making difficult to eliminate them. In addition, mutant p53 possess oncogenic gain of function activities involved in tumor progression, metastasis and metabolism [23, 25, 44]. These tumors must be treated using drugs that specifically inhibit mutant p53. The levels of mutant p53 in several cancer cell lines are high due to its binding to HSP90 that protects mutant p53 from MDM2-mediated ubiquitination and proteasomal degradation. 17-AAG is an HSP90 inhibitor that promotes degradation of mutant p53 [45] with reported efficacy in primary leukemia cells from patients with MM, AML and chronic lymphocytic leukemia (CLL) [46]. We first compared its effect on viability of several MM and AML cell lines with different p53 status, but not in MOLM13 cells as they are very sensitive to 17-AAG due to FLT3 expression [47, 48]. Incubation with 17-AAG for 24 h decreased cell viability particularly in U266 and NB4 cells that harbor mutant p53 (Figure 7A). Moreover, HSP90 inhibition by 17-AAG decreased p53 protein levels in U266 and NB4 cells (Figure 7A, bottom panels), but had no effect in MM1.S cells (wt p53). To decrease 17-AAG toxicity, we then investigated the effect of associating 17-AAG at low concentration with DCA. Like with 17-AAG alone, this drug combination decreased p53 protein level only in leukemic cell lines harboring mutant p53 (U266 and NB4) (Figure 7B). However, lower 17-AAG concentrations were required when used in combination with DCA. Similar results were obtained using primary leukemic cells from two patients with BCL harboring mutant p53 (Figure 7C). Finally, cell numbers and viability were both significantly reduced in primary leukemic cells from three patients with B-CLL harboring mutant p53 upon co-incubation with 17-AAG and DCA (Figure 7D). Cell death, caused probably by apoptosis, was increased in tumor cell lines expressing mutant p53 (Figure 7E).

## DISCUSSION

**Figure 8:**
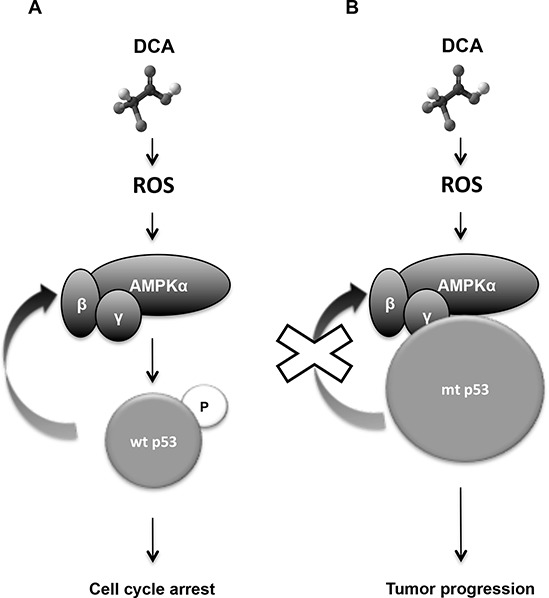
Diagram describing the mechanism of how DCA efficacy depends on p53 status **A.** DCA activates AMPK/p53 pathway in response to oxidative stress. Wt p53 is phosphorylated and transcriptional activated by AMPKα leading to G1 cell cycle arrest and inhibition of glycolysis. p53 also increases *AMPKβ* gene expression, reinforcing the AMP-activated protein kinase (AMPK) response. **B.** DCA doesn't stop proliferation in absence of p53 or presence or mtp53. mtp53 gain of functions could be involved in tumour progression, drug resistance and inhibition of certain genes such as AMPK.

Here, we show that DCA efficacy in several hematological cancers depends on their p53 status. Targeting tumor cell metabolism with DCA leads to p53 accumulation, increase in p53 transcriptional activity and p53-dependent accumulation of cells in G1 phase only in leukemic cell lines and primary leukemic cells harboring wt p53 (Figure 8).

DCA induces p53 phosphorylation through AMPK, thus enhancing p53 effects on cell proliferation and metabolism. In addition, p53 increases transcription of AMPKβ, the regulatory subunit of AMPK. The AMPK/p53 positive feedback loop was previously described [19, 21] and our results support the finding that p53 activation by metabolic stress is activated by AMPK [49]. Hence, AMPK activation is required for p53-mediated growth suppression and metabolic adaptation in response to DCA treatment.

Forcing leukemic cells to perform OXPHOS by glucose deprivation and causing glutaminolysis have similar effects as DCA treatment. Based on this result, we suggest that the activation of the AMPK/p53 pathway by DCA is the consequence of a metabolic change from aerobic glycolysis to OXPHOS. This could be clinically relevant because other drugs with similar metabolic effects as DCA could elicit their effects through the mechanism described in this study [1, 3]. Activation of p53 by DCA not only promotes cell cycle arrest, but could also cause further inhibition of glycolysis. Recent studies have shown that p53 can stimulate energy metabolism of cells through the activation of genes, such as *GLS2* [38, 50] and *SCO2* [51]. SCO2 is an essential regulator of the cytochrome c oxidase complex (mitochondria complex IV) and is induced by p53 to increase OXPHOS [51]. GLS2 stimulates oxidative energy metabolism by increasing α-ketoglutarate production, mitochondrial respiration and linked ATP generation. It has been proposed that GLS2 could also inhibit tumor cell growth, thus contributing to p53 tumor suppressor role [38, 50]. In addition, GLS2 has an anti-oxidant function by decreasing ROS levels to protect cells from oxidative stress and death [38]. DCA promotes mitochondrial OXPHOS, which is the main source of endogenous ROS in the cells. ROS also promotes AMPK phosphorylation and activation [20]. We demonstrated that ROS generated by OXPHOS are, at least partially, responsible for the activation of the AMPK/p53 pathway (Figure 8). ROS-mediated p53 activation could then induce the anti-oxidant response through GLS2 expression up-regulation. Furthermore, loss of p53 or the presence of mutant p53 is associated with increased intracellular ROS production that contributes to accelerate tumor progression [38, 39]. This could explain the observed high basal ROS level in p53^−/−^ and especially in mutant p53 cell lines compared to wt p53 cell lines. However, we do not rule out that other mechanisms could contribute to AMPK activation, such as the increase of the AMP/ATP ratio in response to glycolysis inhibition [52].

For our study, we have used DCA concentrations similar to other groups; however the clinical concentration in DCA-treated patient is unclear. The half-life of DCA is <1 hour: it is not detectable in patients during the initial treatments that can last the first 2 to 3 months [8, 9]. DCA inhibits its own metabolism and serum concentrations increase, eventually reaching a plateau, with plasma concentrations around 0.3 mM [8]. Michelakis et al gave 50 mg/Kg/day of DCA to patients and obtained similar values 0.44 ± 0.16 mM [9]. On average this could give a DCA blood concentration of 5 mM. However, the ultimate destination of the DCA that was not in blood was unknown. In this manuscript we have observed that DCA concentrations of 1 mM can induce some metabolic responses but not all. Consistent results were obtained with concentrations between 5 and 20 mM. But it is important to note that we treat cells for shorter periods of times (3 days) than those used in clinic that can last several months or even years. Hence, without knowing where the DCA is *in vivo* and the half-life *in vitro* we can not compare both situations.

We found that DCA mainly inhibit proliferation rather than inducing apoptosis in tumors with wt p53. This is in agreement with results in other cell lines in which analysis of cells incubated with DCA did not highlight induction of apoptosis [16]. DCA did not have a significant effect on the proliferation of cell lines or primary cells with mutant p53 or lacking p53, although we observed (data not shown) that higher DCA doses and longer incubation times induced cell death in p53−/− cells, as previously described [16].

At high doses DCA can induce neurological complications in humans [14]. To avoid these toxic effects, we propose to associate DCA with standard, anti-leukemic drugs, such as doxorubicin and vincristine. These drugs are widely used to treat lymphoma, myeloma and leukemia. Unfortunately, many tumors are resistant or become resistant to these drugs, leading to treatment failure or relapse. We found that extremely low, non-toxic concentrations of doxorubicin and vincristine synergized *in vitro* with DCA to reduce viability of primary leukemic cells. Our results show that these drug combinations are optimal to induce p53 accumulation and p53-dependent cell cycle arrest and apoptosis. In addition, our results show that DCA and doxorubicin synergize to inhibit proliferation of human p53^+/+^ AML cells *in vivo*. However, this is transitory and additional experiments with longer incubation times are required to determine whether this treatment can effectively and durably block tumor progression.

Conversely, all tumor cell samples from patients with mutant p53 (*n* = 4) were resistant to DCA alone or in combination with genotoxic drugs, suggesting that the response to this treatment could be associated with the tumor p53 status. In these patient samples and in p53 mutant cell lines, co-treatment with DCA and 17-AAG was clearly more effective in inducing apoptosis than single-drug treatments. 17-AAG promotes mutant p53 degradation by the ubiquitin proteasome pathway [45]. The combination of 17-AAG and DCA strongly reduced p53 protein levels in p53 mutant cells and increased apoptosis of tumor cells. At the present, it remains unclear how DCA and 17-AAG synergize to reduce mutant p53 protein level. Further studies are necessary to elucidate this effect.

Mutations in p53 are not equivalent to p53 loss. Mutant p53 acquires new functions to drive tumor cell migration, invasion and metastasis formation [53] and it may also have opposite functions to wt p53 in the control of metabolism. For instance, mutant p53 promotes aerobic glycolysis by induction of type II hexokinase (*HK2*) gene expression [54]. Our data suggest that mutant p53 may limit the efficacy of DCA in blocking tumor progression and are consistent with recent studies showing that mutant p53 promotes cancer cell growth and metabolism by inhibiting AMPK activation [25]. This provides a possible mechanism for the observed resistance of mutant p53 tumor cells to DCA treatment. By promoting mutant p53 degradation with the HSP90 inhibitor 17-AAG we can increase DCA potency as a cancer therapeutic drug. And *vice versa*, DCA can facilitate the clinical use of 17-AAG by decreasing the required concentrations and consequently its side effects. This is clinically relevant because, in hematological malignancies, p53 mutation frequency increases with tumor progression to more aggressive or advanced stages and it is strongly associated with chemo-resistance and poor prognosis [26–29, 55].

In summary, we provide a new mechanism of action of DCA through the AMPK/p53 pathway and provide the proof of principle and the proof of mechanism that depending on the p53 status, different combinatorial therapies that include metabolic drugs, such as DCA, will provide better treatment with decreased side effects. Furthermore, our finding that suppressing mutant p53 could specifically induce apoptosis in hematological cancers carrying p53 mutations, offers a potential new therapeutic approach in relapsed patients.

## MATERIALS AND METHODS

### *In vivo* mouse experiments

*In vivo* experiments were carried out using 6 to 8 weeks/old NSG mice. Mice were bred and housed in pathogen-free conditions in the animal facility of the European Institute of Oncology–Italian Foundation for Cancer Research (FIRC), Institute of Molecular Oncology (Milan, Italy). All animal experiments were carried out in accordance with national and international laws and policies. For induction of acute leukemia in mice, 1 million AML cells were injected intravenously (i.v.) through the lateral tail vein in non-irradiated mice. At day 80, when human cells reached 1% in blood, mice were separated in four groups of four mice: control, doxorubicin (1.5 mg/kg, 3 doses i.v. from day 5 to day 7), DCA (50 mg/kg, 1 dose/day by gavage, starting at day 1 for 16 consecutive days) and DCA+doxorubicin as described above. At days 100, 120 and 140 post-graft, peripheral blood was collected from the tail vein and the percentage of human AML cells was analyzed by flow cytometry, as previously described [42].

### Cell cultures

AML (MOLM13, NB4 and HL60) and MM (MM1.S and U266) cell lines were cultured as previously described [41]. MOLM13 and HL60 cells were kindly provided by Dr. Bossis from the IGMM institute (Montpellier, France). MOLM13 and MM1.S cells harbor wt p53, NB4 and U266 cells carry mutant p53 (R248Q and A161T, respectively) and HL60 is a p53^−/−^ cell line. p53^+/+^ and p53^−/−^ HCT116 cells were cultured in low glucose (5 mM) DMEM medium supplemented with 10% FBS. Bone marrow and peripheral blood samples were obtained from patients with different hematological diseases and healthy donors after informed consent. Cells were purified by Ficoll-Hypaque (Sigma) density-gradient centrifugation. Primary cells were seeded in RPMI 1640 medium supplemented with 10% FBS, containing 10 ng/ml IL-6 for MM (CD38^+^CD138^+^), 10 ng/ml IL-10 for BCL (CD5^+^CD19^+^) or nothing for B-CLL (CD19^+^CD20^+^) samples. Cells were grown at 37°C and 5% CO_2_ in a humidified atmosphere. In summary all samples were grown in the approximately physiological glucose concentration of 5 mM.

### Reagents, siRNAs and transfection

DCA was purchased from Santa Cruz, 17-AAG was from Selleck, doxorubicin and vincristine were obtained through the University Hospital of Montpellier (CHU Montpellier). All siRNA duplexes used for AMPKα1 knockdown were ON-TARGETplus modified siRNAs (Dharmacon). p53 siRNA was a gift from Dr Xirodimas and it was ON-TARGETplus SMARTpools (mixture of 4 siRNA) from Dharmacon. In HCT116 cells, transfection of 30–50 nM siRNAs was carried out using Lipofectamine RNAiMAX (Invitrogen) in Opti-MEM (Invitrogen), according to the manufacturer's instructions. MOLM13 cells (2 × 10^6^ in 100 μl) were transfected with 300 nM AMPKα1 siRNA, or control siRNA by electroporation using the Gene Pulser Xcell™ Electroporation system (Bio-Rad). Cells were harvested 24 to 72 h post-transfection.

### Cell proliferation, viability and apoptosis

Cell viability and cell numbers were determined using the Muse^®^ Cell Analyzer (Millipore) or the trypan blue exclusion method, as indicated. Analysis of apoptotic cells (Annexin V assay) was performed using the Muse^®^ Cell Analyzer. In some experiments cells were incubated in RPMI 1640 without glucose and supplemented with 10 mM galactose and glutamine (final concentration: 4 or 6 mM) to force cells to perform OXPHOS [40, 56].

Cell cycle analysis was performed using the Click-iT EdU flow cytometry assay kit (Invitrogen, Molecular Probes). For ROS detection, cells were incubated in PBS containing 10 μM DCFH2-DA or CellROX^®^ Deep Red Reagent (Invitrogen, Molecular Probes) for 30 min, and then washed with PBS and immediately analyzed by flow cytometry.

The viability of primary tumor cells was determined using the fluorescent marker 7-amino-actinomycin D (7-AAD) (Beckman). Different antibodies (Beckman) against surface markers were used to identify tumor cells: anti-CD5-PB, -CD19-PE and -CD20-AlexaFluor750 for cells from patients with B-CLL, anti-CD38-PE-Cy5.5 and -CD138-APC for cells from patients with MM, anti-CD14-FITC, -CD33-APC and -CD34-PE for cells from patients with AML, anti-CD33-APC and CD34-PE for cells from patients with myelodysplastic syndrome (MDS), anti-CD10-APCAlexaFluor750, -CD19-PE or -CD19-PE and -CD20-AlexaFluor750 for cells from patients with B-cell lymphoma, and anti-CD3-APC for T lymphocytes from healthy donors. 1 × 10^6^ cells were incubated with the corresponding antibodies in PBS supplemented with 2.5% FBS at 37°C for 20–30 minutes. Cells were then washed, resuspended in 200–250 μl PBS with 2.5% FBS and analyzed using a Gallios™ flow cytometer (Beckman Coulter). Data were analyzed with the Kaluza software.

Number of cells means number of live cells and viability is the number of live cells counted divided by total cell counted (alive and dead cells). A decrease of number of live cells could be due to a cell cycle arrest and/or a decrease of viability. A decrease in viability is due to an increase of cell death (e.g., apoptosis or necrosis).

### Western blot analysis

Primary antibodies against phospho-AMPKα (Thr172), AMPKα, AMPKβ, phospho-ACC (Ser79), ACC, phospho-p53 (S15 and S46), p21 (12D1) and β-actin were from Cell Signaling Technology. The anti-p53 antibody (DO-1) was a gift from Dr Xirodimas. Cell extracts were lysed in 2 × SDS sample buffer. Proteins were resolved by SDS-PAGE and transferred to nitrocellulose or PVDF membranes using the Trans-Blot^®^ Turbo™ Transfer System (Bio-Rad). Peroxidase-coupled anti-mouse and anti-rabbit secondary antibodies were used at a dilution of 1:10, 000 (Sigma). Bound antibodies were detected by enhanced chemiluminescence (Millipore) or using the Supersignal West Dura extended duration substrate (Pierce). Chemiluminescent detection was carried out using the Molecular Imager Gel Doc XRS system (Bio-Rad). Band quantification was performed using the Image lab software (Bio-Rad). The P-ACC/ACC ratio is calculated by given the value of 1 to untreated cells for each independent cell line and reporting to this value the P-ACC/ACC ratio in stimulated cells.

### RT-PCR and DNA sequencing

Total RNA was extracted using NucleoSpin RNA isolation columns (Macherey-Nagel), reverse transcription was carried out using random primers. Quantitative PCR was performed as described previously [57] with SsoADV SYBR Green qPCR SuperMix (Biorad) and a CFX Connect™ Real-Time qPCR machine (Biorad) with AMPKα1, AMPKβ1, GLS2, MDM2 (P2), p21, SCO2 and actin primers. All samples were normalized to β-actin mRNA levels. Primer sequences are listed in Supplementary Materials and Methods. p21, GLS2 and PUMA primers were previously described [50]. For p53 sequencing, 1 μg of RNA was reverse-transcribed using random primers. One twentieth of the cDNA was amplified by nested PCR using the appropriate primers [58]. PCR products were separated on agarose gels. The major bands were excised and sequenced. To determine the tumor p53 status, the entire open reading frame was sequenced by Eurofins MWG Operon with the primers E67F (5′-TTGCGTGTGGAGTATTTGGAT-3′) and MP9ER (5′-TCTCCCAGGACAGCACAAACACG-3′).

### Oxygen consumption

Mitochondrial oxygen consumption assays were performed using the high-resolution respirometry system Oxygraph-2k (Oroboros, Austria) as described previously [59].

### Statistical analysis

Statistical analysis was performed using the Student's *t* test: *p < 0.05; **p < 0.01; ***p < 0.001. Values were expressed as the mean ± the standard error of the mean (SEM).

## SUPPLEMENTARY MATERIALS AND METHODS FIGURES AND TABLE


