# Supplementary material for: The presence of wild type p53 in hematological cancers improves the efficacy of combinational therapy targeting metabolism
Source: Oncotarget. 2015 Jul 30;6(22):19228–45. doi: 10.18632/oncotarget.4653 (PMC4662487; doi:10.18632/oncotarget.4653)
Supplement: Supplementary file 1 [file oncotarget-06-19228-s001.pdf]

SUPPLEMENTARY MATERIALS AND METHODS

Sequence of the primers used for PCR analyses and catalogue numbers of the ONTARGET plus siRNAs (Dharmacon) used for AMPKα knockdown. A single siRNA complementary to the target was used for the experiments.

Mdm2 forward 5′-CGGACGCACGCCACTT-3′
Mdm2 reverse 5′-CAGTAGGTACAGACATGTTGGTATTGC -3′
p21 forward 5′-GGCGGCAGACCAGCATGACAGATT- 3′
p21 reverse 5′-GCAGGGGGCGGCCAGGGT-3′
GLS2 forward 5′- TGCCTATAGTGGCGATGTCTCA-3′
GLS2 reverse 5′-GTTCCATATCCATGGCTGACAA-3′
SCO2 forward 5′-GCAGCCTGTCTTCATCACTGTGGACC-3′
SCO2 reverse 5′-CCGCACACTGTCTGAGATCTGCTC-3′
AMPKβ forward 5′-CCTCACCAGAAGCCACAATA-3′
AMPKβ reverse 5′-AGCTGGCTGGTTACTATGGG-3′
PUMA forward 5′-CCTGGAGGGTCCTGTACAATCT-3′
PUMA reverse 5′-GCACCTAATTGGGCTCCATCT-3′
Actin forward 5′-GAGGGAAATCGTGCGTGACA-3′
Actin reverse 5′-AATAGTGATGACCTGGCCGT-3′

siRNA

AMPKα (1): 007675–05
AMPKα (2): 007675–06

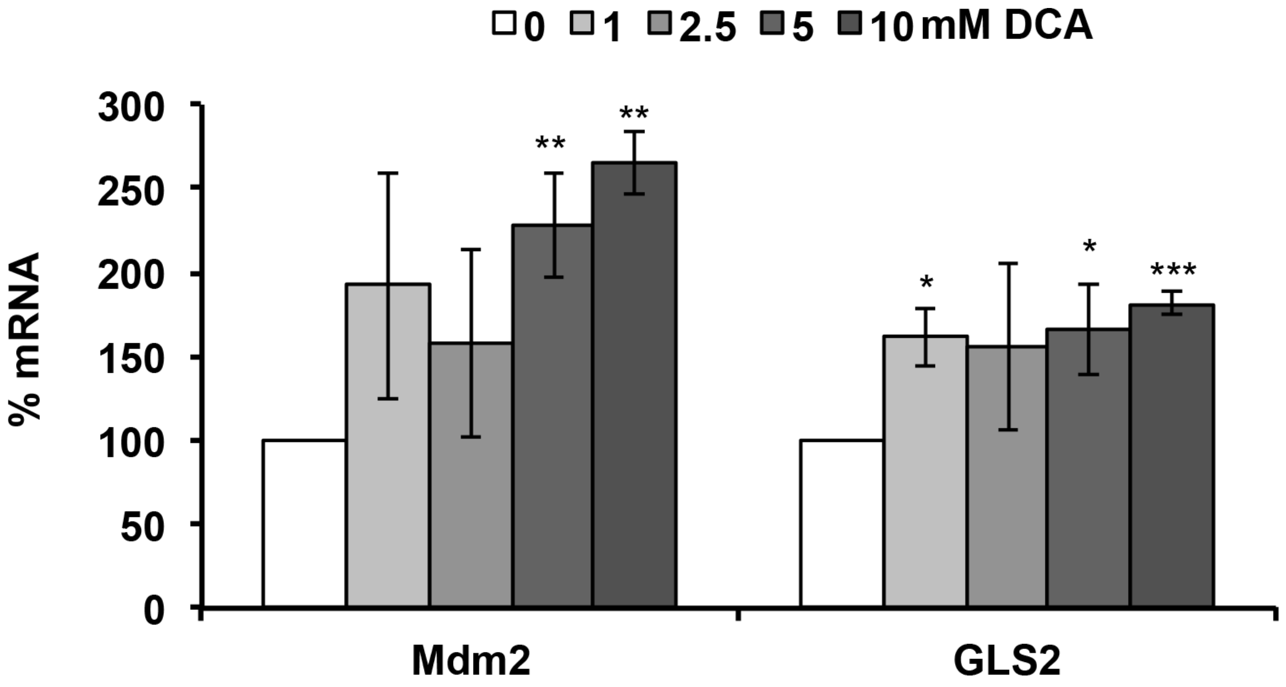

Supplementary Figure S1: Related to Figure 1. Low doses of DCA activates p53 transcription activity. MOLM13 cell line were treated with low concentration of DCA for 24 h. mRNA levels of Mdm2 and GLS2. Values are the mean ± SD of two independent experiment *$p < 0.05$; **$p < 0.01$; ***$p < 0.001$.

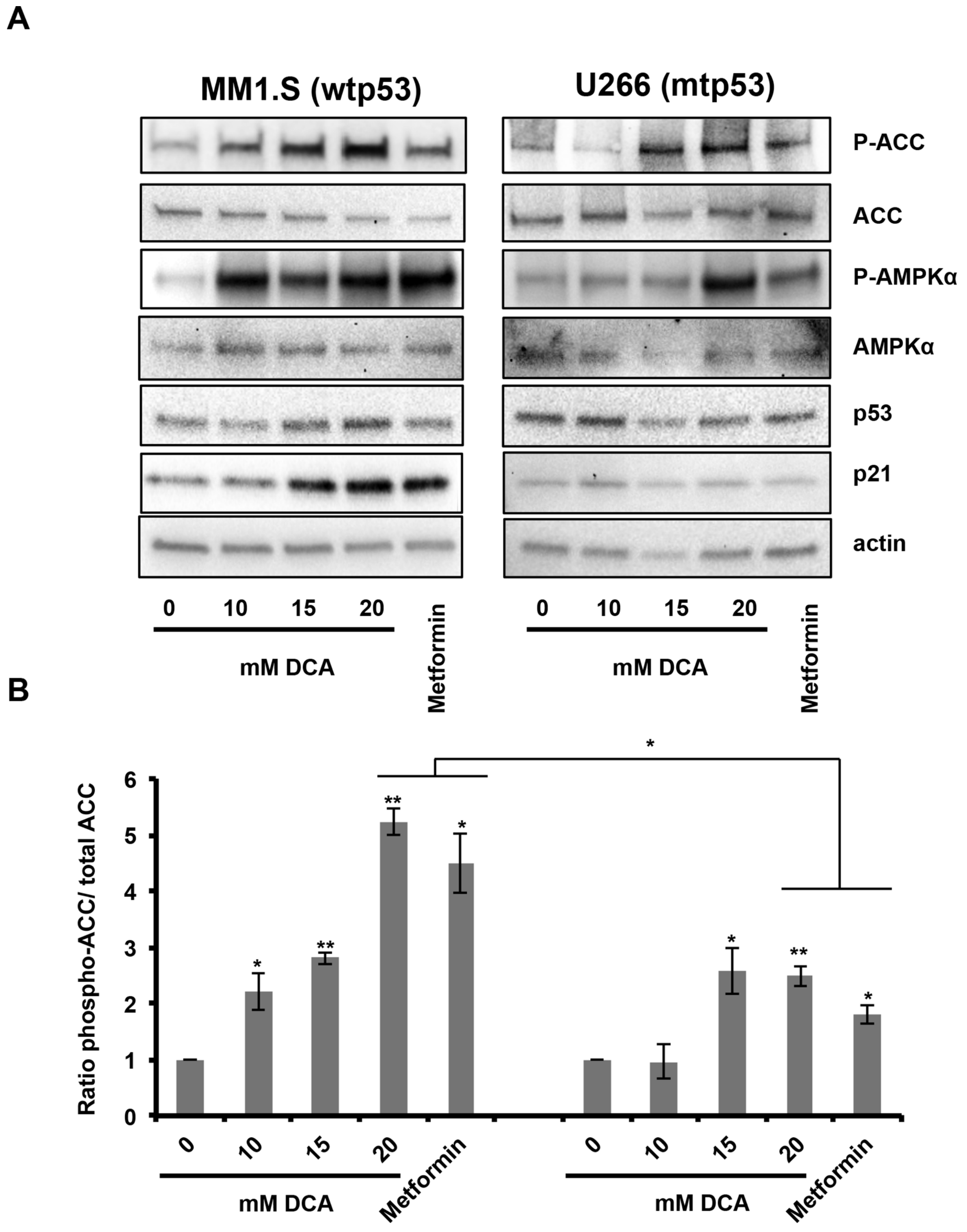

Supplementary Figure S2: Related to Figure 2 and 3. DCA induces the activation of the AMPK/p53 pathway. **A.** MM cells were incubated with the indicated concentrations of DCA for 24 hours. DCA increased the protein expression of P-AMPK, P-ACC, p53 and p21 in MM1.S cells that carry wild type p53 (wtp53). Phosphorylation of AMPK and ACC required higher doses of DCA in the U266 cell line (mutant p53, mtp53) and no increase of p53 and p21 protein levels was detected. **B.** Graph representing the ratio between phosphorylated ACC and total ACC protein levels. Bands were quantified using the Image lab software. Values are the mean ± SD of two independent experiments.

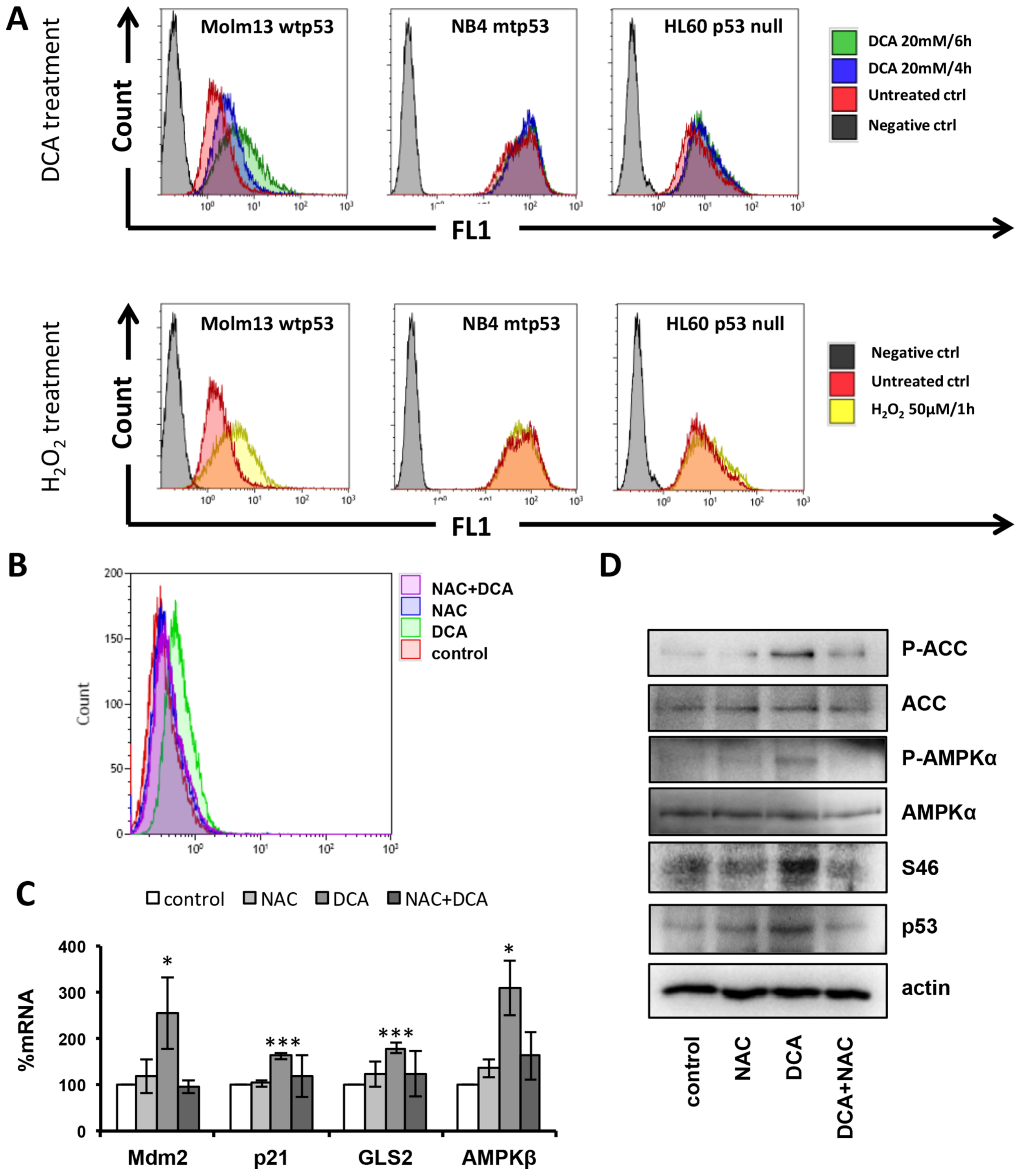

Supplementary Figure S3: Related to Figure 1 and Figure 2. A. Effect of DCA on ROS production in AML cells. AML cells were incubated with 20 mM DCA for 4 and 6 hours or with H_2O_2 for 1 hour. For ROS detection, cells were incubated with 10 µM DCFH2-DA for 30 min before flow cytometry analysis. DCA and H_2O_2 treatment increased ROS production in all AML cell lines, but ROS production was higher in MOLM13 cells (wtp53). **B.** AMPK/p53 activation was inhibited by blocking DCA-ROS production (CellROX® Reagent) using NAC (N-acetyl-L-cysteine).**C.** and **D.** 1mM NAC blocks DCA-induced ACC and p53 phosphorylation as well as Mdm2, p21, GLS2 and AMPKβ mRNA expression. Values are the mean ± SD of two independent experiments.

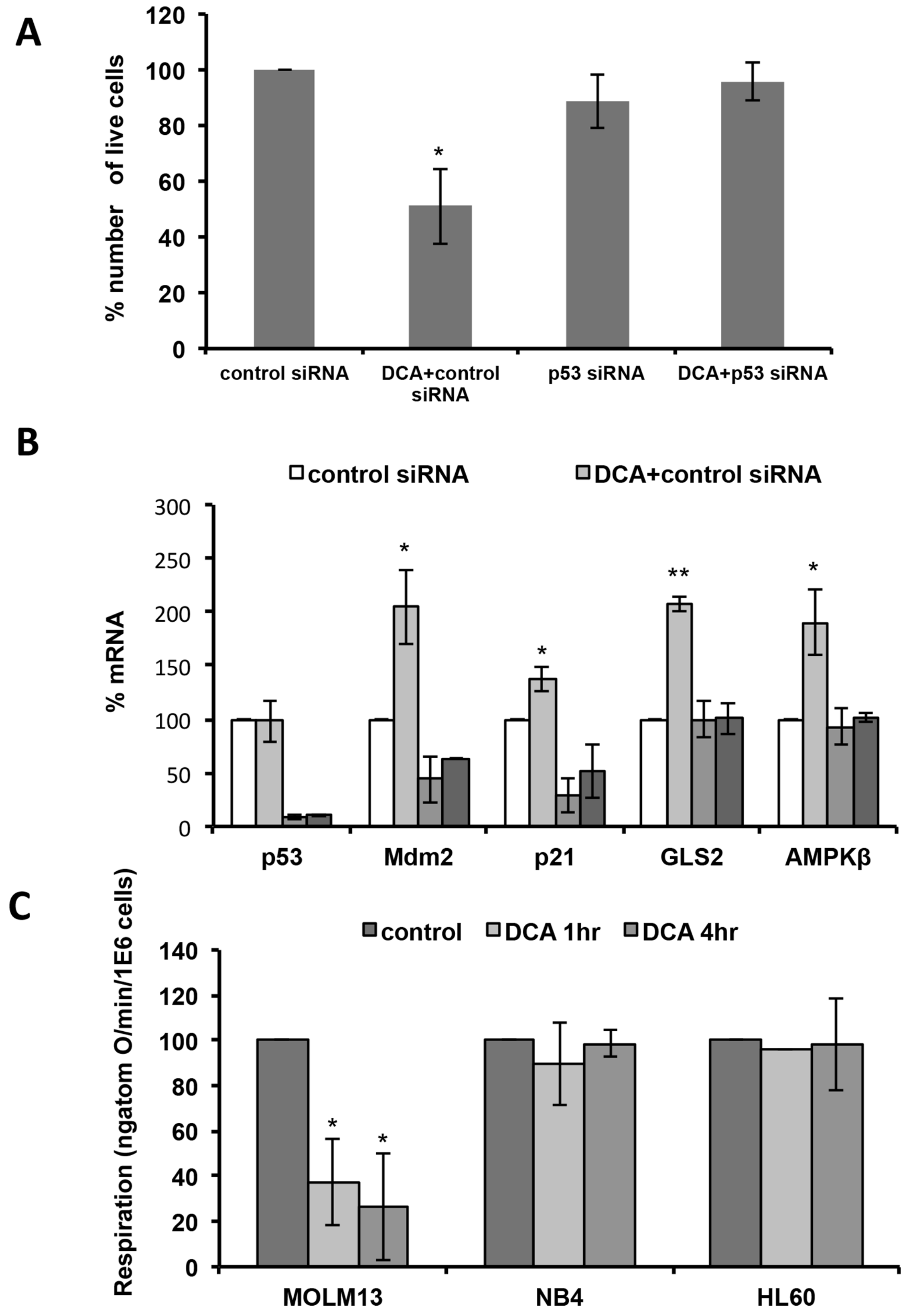

Supplementary Figure S4: Related to Figure 3. Knockdown of p53 abolishes the effect of DCA on cell proliferation and Mdm2, p21, GLS2 and AMPKβ mRNA expression in HCT116+/+ cell line. HCT116+/+ cells were transfected with 30 nM control or p53 siRNA. After 24 hr transfection, 10 mM DCA was added for another 24 hr. **A.** A reduction of alive cells was only observed in the cells transfected with control siRNA and treated with DCA. Number of cells were counted using the Muse™ Cell Analyzer. **B.** mRNA levels of the indicated genes were analysed by RT-qPCR. **C.** Effect of DCA on oxygen consumption in AML cell lines. A decrease oxygen consumption was observed in MOLM13 cell line (wt p53) after DCA treatment (10 mM) for 1 and 4 hours whereas there was not effect in NB4 and HL60 cells. Values are the mean ± SD of two independent experiments *$p < 0.05$; **$p < 0.01$; ***$p < 0.001$.

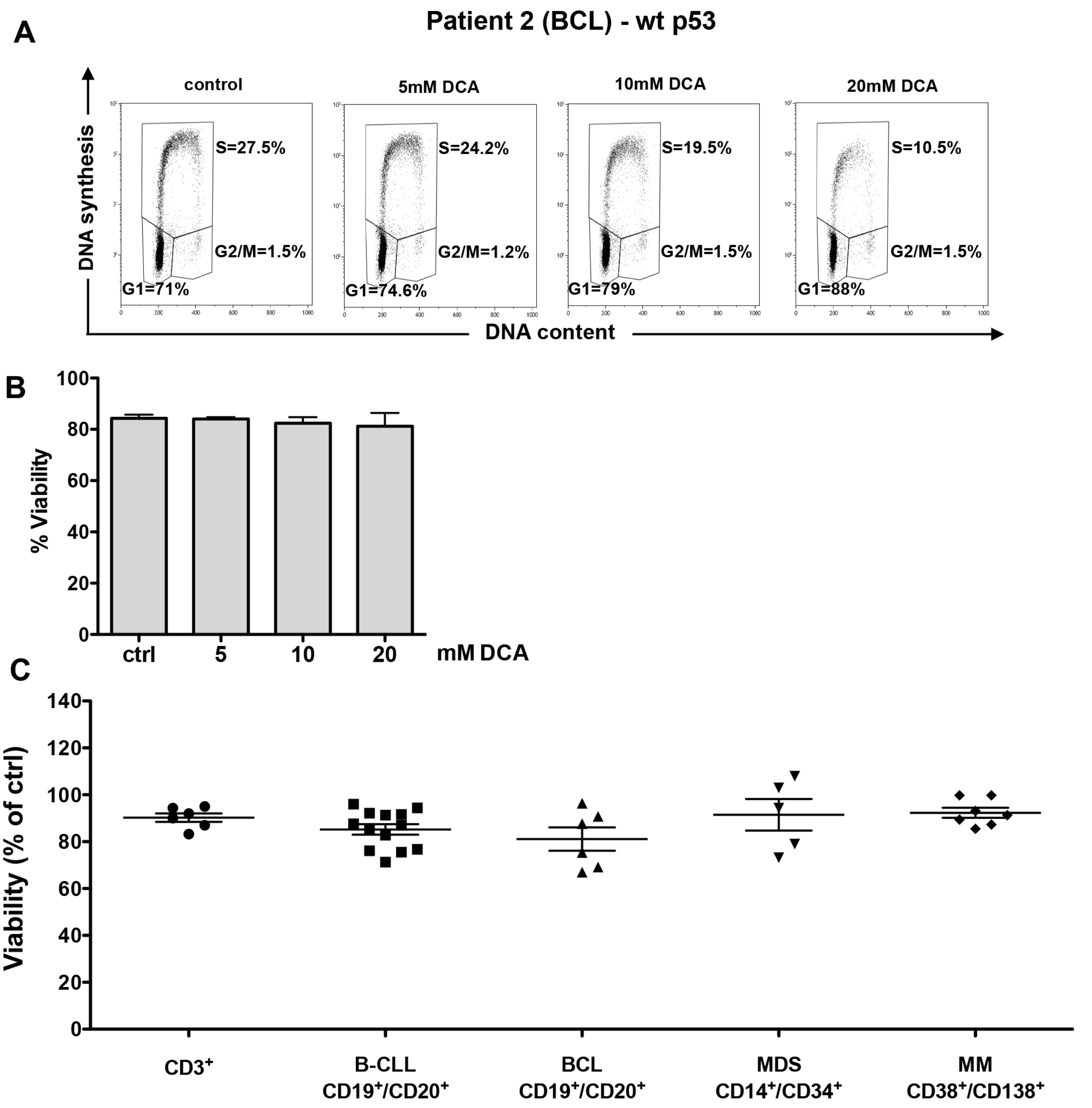

Supplementary Figure S5: Related to Figure 1. Effect of DCA on proliferation and viability of primary tumor cells. **A.** DCA causes G1 cell cycle arrest in primary tumor cells. Tumor cells obtained from Patient 2 (B-cell leukemia, BCL) were cultured in the presence of different concentrations of DCA for 48 h. Then, cells were pulse-labeled with EdU and harvested for cell cycle analysis. **B.** DCA does not affect viability of primary tumor cells. Patient 2 primary BCL cells were incubated with the indicated concentrations of DCA for 48 h. Cell viability was determined by 7-AAD staining and flow cytometry (values are the mean ± SD of three independent experiments performed in triplicate). **C.** Primary tumor cells from 13 patients with B-cell chronic lymphocytic leukemia (B-CLL) ($CD19^+/CD20^+$), six patients with B-cell lymphoma (BCL, $CD19^+/CD20^+$), five patients with myelodysplasia (MDS, $CD14^+/CD34^+$) and seven patients with MM ($CD14^+/CD34^+$) (all wt p53) were treated with 10 mM DCA for 48 h before 7-AAD staining and analysis by flow cytometry. $CD3^+$ cells from healthy donors (*n* = 6) were used as control. Values are the mean ± SD of one experiment performed in triplicate.

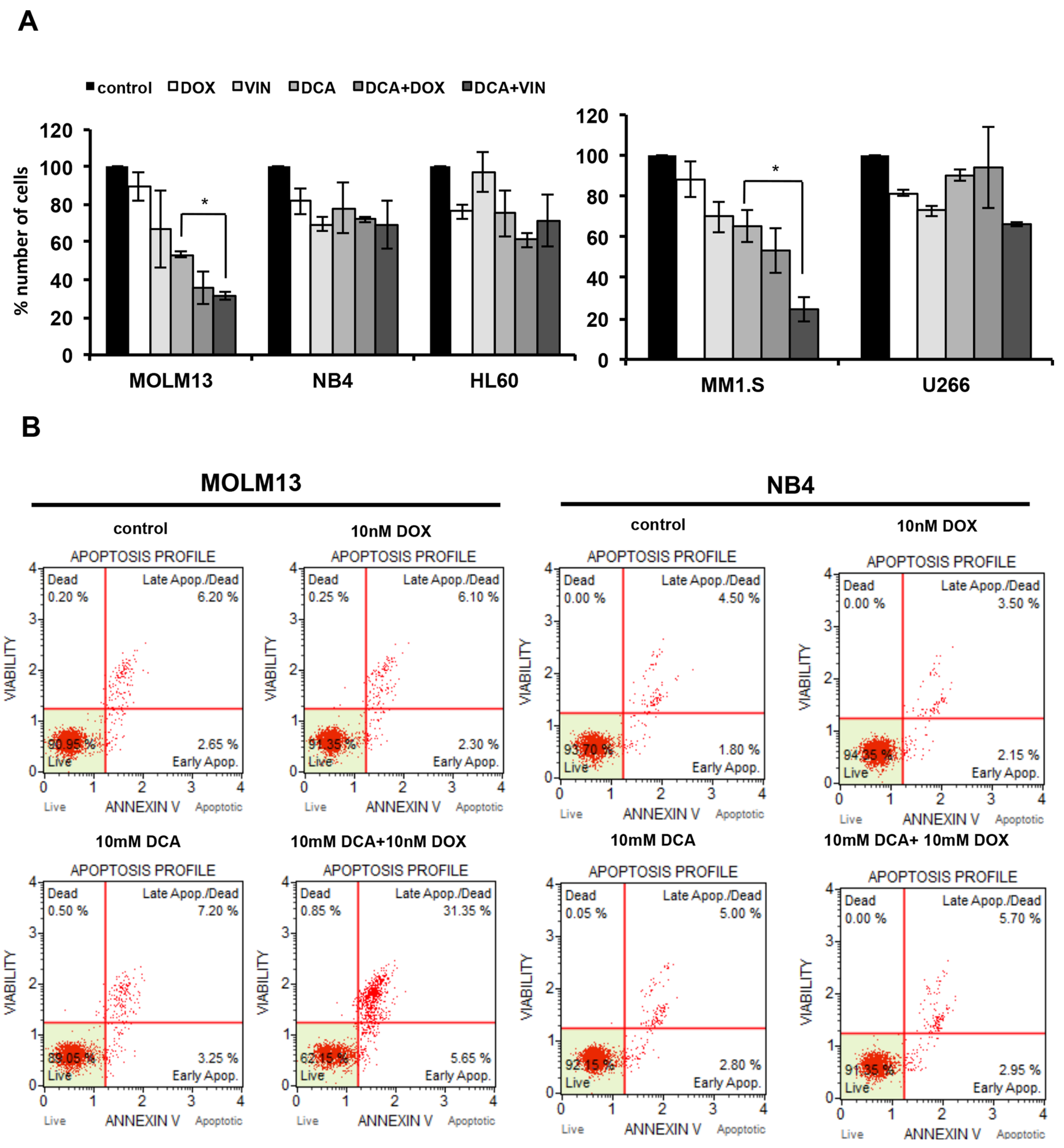

Supplementary Figure S6: Related with Figure 6. Additive effect of DCA and doxorubicin or vincristine. **A.** Co-treatment with DCA and doxorubicin (DOX) or vincristine (VIN) at low dose reduced the number of alive cells in wt p53 but not in p53 mutant AML and MM cell lines. Cells were incubated with 10 mM DCA overnight and the next day 10 nM doxorubicin or 10 nM vincristine was added for 24 hours. The number of viable cells was determined by using the Trypan blue exclusion method. Values are the mean ± SD of two independent experiments **B.** Co-treatment with DCA and DOX increases the number of apoptotic cells in MOLM13 cells (wt p53) but not NB4 cells (mutant p53). Annexin V assay was performed using the Muse® Cell Analyzer.

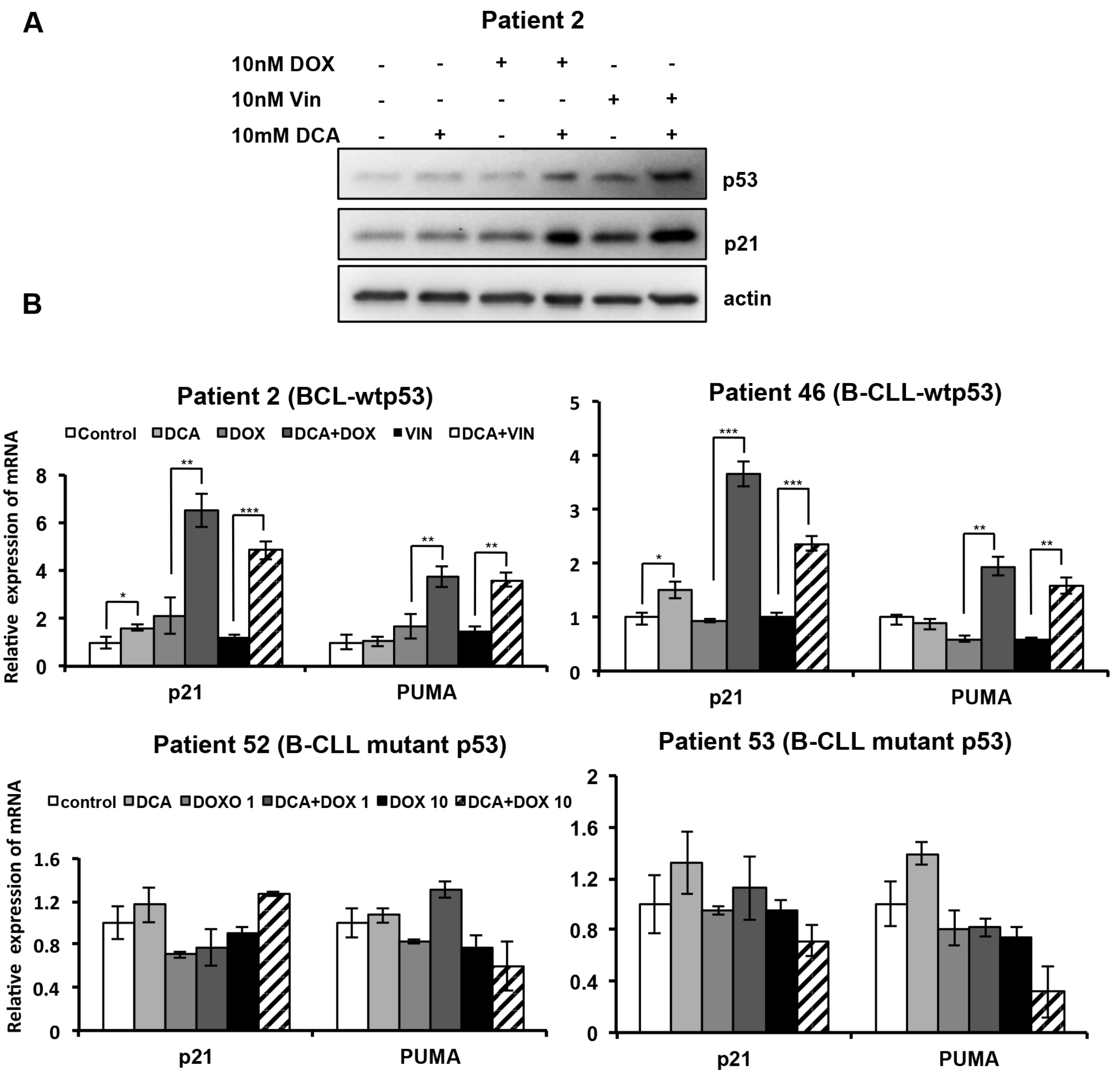

Supplementary Figure S7: Related with Figure 6. Synergistic effect of DCA and doxorubicin (DOX) or vincristine (VIN) in primary cells from Patient 2 (BCL). **A.** Co-treatment with DCA and low doses of DOX or VIN. Cells were pre-incubated with 10 mM DCA overnight and the next day 10 nM DOX or 10 nM VIN was added for 24 hours. p53 and p21 expression were revealed by western blotting. **B.** mRNA levels of p21 and PUMA were analysed by RT-qPCR from patient 2 tumor cells. The wt p53 status of this patient was confirmed by sequencing. Values are the mean ± SD of one experiment repeated in triplicate *$p < 0.05$; **$p < 0.01$; ***$p < 0.001$.

Supplementary Table S1: Cancer type and p53 status of the patients' samples and cell lines used for this study

Patient	Type of cancer	p53 status
P2	BCL	wt
P7	B-CLL	wt
P8	BCL	wt
P10	MDS	wt
P13	MDS	wt
P14	MM	wt
P15	MDS	wt
P18	B-CLL	wt
P19	BCL	wt
P21	B-CLL	mt several point mutations
P22	MM	wt
P25	B-CLL	wt
P28	MM	wt
P33	B-CLL	wt
P36	MDS	wt
P38	BCL	wt
P39 = P52	B-CLL	mt P82L
P40	B-CLL	wt
P43	B-CLL	wt
P44	MDS	wt
P45	B-CLL	wt
P46	B-CLL	wt
P49	MM	wt
P51	MM	wt
P53	B-CLL	mt, early stop codon
P54	B-CLL	wt
P55	MM	wt
P56	MM	wt
P67	B-CLL	wt
P69	BCL	wt
P73	BCL	wt
P81	B-CLL	wt
P90	B-CLL	mt G360R
P99	B-CLL	wt
P1A	B-CLL	wt
P2A	B-CLL	wt

(*Continued*)

Cell line	Type of cancer	p53 status
MOLM13	AML	wt
NB4	AML	mt R248Q
HL60	AML	null
MM1.S	MM	wt
U266	MM	mt A161T
HCT116 +/+	Colorectal cancer	wt
HCT116 −/−	Colorectal cancer	null
